# Metal Oxides-Based Semiconductors for Biosensors Applications

**DOI:** 10.3389/fchem.2020.00354

**Published:** 2020-05-19

**Authors:** Ionel Şerban, Alexandru Enesca

**Affiliations:** Product Design, Mechatronics and Environmental Department, Transilvania University of Brasov, Brasov, Romania

**Keywords:** metal oxides, semiconductors, nanostructures, energy bands, biosensors

## Abstract

The present mini review contains a concessive overview on the recent achievement regarding the implementation of a metal oxide semiconductor (MOS) in biosensors used in biological and environmental systems. The paper explores the pathway of enhancing the sensing characteristics of metal oxides by optimizing various parameters such as synthesis methods, morphology, composition, and structure. Four representative metal oxides (TiO_2_, ZnO, SnO_2_, and WO_3_) are presented based on several aspects: synthesis method, morphology, functionalizing molecules, detection target, and limit of detection (LOD).

## Introduction

Biosensors represent key components in medical care, environmental processes, energy efficient systems, food safety, chemical, and agricultural industries. The necessity of using continuous onsite monitoring with flexible and reliable characteristics have recommended biosensors as an efficient tool for rapid measurement and analysis. Adapting the biosensors materials to various applications (quality control, screening methods, safety equipment, environmental evaluation) represents an important research topic with difficult challenges to overcome. In the last decade there were many papers presenting materials such as photonic crystals (Hocini et al., 2019), polymers (Gupta et al., 2020), graphene (Yuan et al., 2019), metals (Rezaei et al., 2019), transition metal dichalcogenties (Wang et al., 2017), and metal organic frameworks (Osman et al., 2019) as suitable for biosensors applications. Some of these materials require significant improvement regarding morphologic optimization, chemical stability, compatibility with different biomolecules, and increase of LOD.

A particular case is represented by MOS materials. These have a high potential to become highly competitive materials in the biosensors market, based on their morphologic versatility (Song et al., 2020), chemical stability (Hernández-Cancel et al., 2015), physicochemical interfacial properties (Scognamiglio et al., 2019), and their ability to combine in composite structures (Zheng et al., 2020). Among others, TiO_2_ (Wang M. et al., 2019), WO_3_ (Liu et al., 2015), SnO_2_ (Dong and Zheng, 2014), and ZnO (Zhang et al., 2019) have attracted considerable attention due to their electrochemical sensitive properties (Enesca et al., 2012a) and energy band alignment (Enesca et al., 2012b) suitable for enzyme based biosensors. Another advantage of these materials is represented by a large number of cost effective synthesis methods such as co-precipitation (Dong and Zheng, 2014), sonochemical precipitation (Zhou et al., 2013), thermal oxidation (Li et al., 2010), chemical etching (Liu et al., 2010), polyol (Elahi et al., 2019), hydrothermal (Zhou et al., 2017), or sol-gel (Rathinamala et al., 2019) allowing the formation of various morphologies such as porous quasi-nanospheres (Liu H. et al., 2017), hollow nano-spheres (Santos et al., 2016), nanorods (Dong et al., 2017), nanosheets (Zhang et al., 2020), or flower-like particles (Feng et al., 2018). Additionally, these materials can be combined between them or with others to form tandem heterostructures (Enesca et al., 2015), hybrid structures (Mihaly et al., 2008), or composite structures (Visa et al., 2016) with advanced electrochemical properties which can be adapted to a specific biosensor application.

MOS with multifunctional properties able to monitor molecules from biological systems represent a step forward in the development of more complex autonomous medical decision-making systems. Enzyme-based biosensors containing MOS have several advantages such as: (1) chemical stability in various environments (Zheng et al., 2020), (2) high energy efficiency (Solaimuthu et al., 2020), (3) good sensitivity (Yi et al., 2020), and (4) adaptability to specific working conditions (Han et al., 2019). There are several issues to overcome in order to implement MOS in biosensing applications: organic/inorganic interface compatibility, increasing the carrier charge mobility, decreasing electron-hole recombinations, and finding facile synthesis techniques.

The present mini review represents a synthesis of the recent achievement of the implementation and optimization of MOS used as biosensor components in biological and environmental systems. The paper is focused on various methods of enhancing the metal oxides' sensing characteristics by optimizing parameters such as synthesis methods, morphology, composition, and structure.

## The Mechanism of Enzyme-Based Biosensors

**Figure 1:**
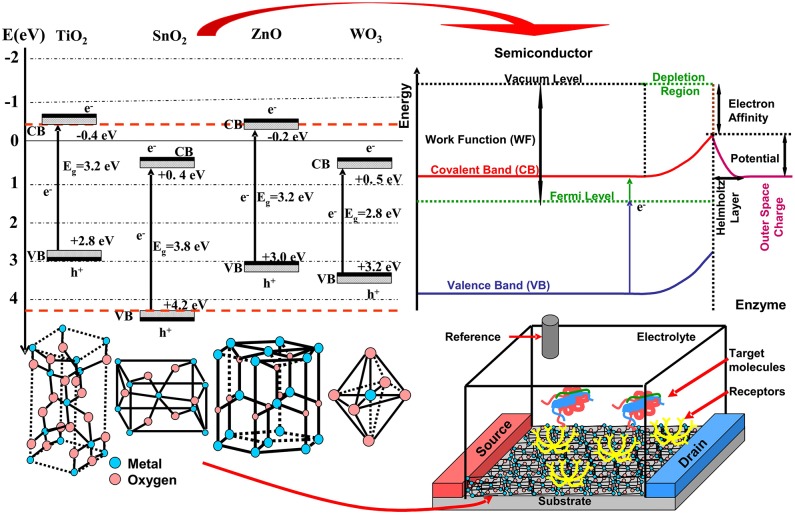
MOS used in biosensors: band energies, crystalline structure, and biosensor configuration.

A biosensor structure (see Figure 1) can be broken down in a biotransducer and its auxiliary signal processing elements (Yin et al., 2018). The biotransducer is made up of a biocompatible layer that has biological recognition entities (enzymes, probe molecules, proteins, etc.) attached to the transducer surface. These entities induce a physicochemical interaction between the target analyte and the transducer, sending signal impulses to the signal processor. An important challenge represents the compatibility between metal oxide inorganic materials with the organic material Wang Q. et al., 2019; Yilmaz et al., 2020. In this sense MOS (i.e., TiO_2_, WO_3_, SnO_2_, ZnO) functionalization in order to increase the compatibility with the organic materials has attracted much interest. The metal oxide semiconductors present some advantages regarding biomolecule immobilizations, such as: (a) high isoelectric point (IEP) which induces electrostatic attraction forces with many lower electrostatic point biomolecules (Ramon-Marquez et al., 2018; Zhao et al., 2019) and (b) morphological versatility exhibited by a high surface area-to-volume ratio characteristic for nanomaterials and favorable for enzyme immobilization, (Fiorani et al., 2019).

Most of the metal oxides materials used in biosensors are wide band gap semiconductors (see Figure 1) consisting of various crystalline structures, unique electrochemical, optical, electronic, gravimetric, pyroelectric, and piezoelectric properties (Huang et al., 2016). Surface potential represents an important property in the biosensor application. The space charge effect is a result of native and imposed semiconductor potential. The bulk chemical composition, crystallization degree, and chemical interaction between semiconductor surface and the analyte will influence the displacement of Fermi energy and induced depletion (Cao et al., 2020). Consequently, the surface potential inducing the space charge double layer is directly dependent on the adsorbed layer characteristics of the electrode surface (Chen et al., 2019). Additionally, some of these materials may exhibit super hydro –phobicity/philicity, self-cleaning and antimicrobial activity. Other factors such as light exposure at certain wavelengths (~380 nm for TiO_2_ and ZnO, ~320 nm for SnO_2_ and ~443 nm for WO_3_) induce an increase of charge carrier mobility. During the light irradiation, oxygen vacancies are formed at the semiconductor surface, which can forward develop oxidative species (Ge et al., 2019).

An important advantage in the metal oxides semiconductor functionalization for biosensor application is represented by the low toxicity and low probability of negative interference with the common pharmaceutical compounds (Soldatkina et al., 2018). The major functionalization methodologies are based on covalent interactions (Feizabadi et al., 2019) but non-covalent interactions have been used as well (Ortiz et al., 2019). The covalent conjugation can be done using different molecules such as dimercaptosuccinic acid (DMSA), 1-ethyl-3-(3-dimethylaminopropyl) carbodiimide), (EDC), N-(15-carboxypentadecanoyloxy) succinimide, 16-(2-pyridyldithiol) hexadecanoic acid, etc. During the functionalization the MOS became more stable and reduced the nanoparticles aggregation, (Xu et al., 2020). After functionalization many analytes can be used as detecting materials for: urea, immunoglobulin, DNA, RNA, dopamine, cancer cells, viruses, etc.

The interactions between the bio-transducer and the analytes will alter the physico-chemical surface semiconductor characteristics. The surface potential, impedance or current characteristics can be correlated with the specific chemical stimuli induced by the analytes (Yoo et al., 2019). Various techniques are based on these characteristics, such as cyclic voltametry, impedimentric, differential pulse voltametry, etc.

The fidelity of the results from such a biosensor are however heavily influenced by the environmental factors of the experiment, such as temperature, humidity, pH, presence of oxygen, and foreign organic compounds, all of which can affect the stability of the interface. The applications of such biosensors spread across all domains, depending on the possible interactions between the immobilized biomolecules (enzymes, antibodies, DNA) and the analytes of interest.

## Metal Oxide Semiconductors for Enzyme-Based Biosensors

There are various MOS's used in biosensor applications. The majority part use mono-component semiconductors but there are many papers (Oh et al., 2013; Kao et al., 2015) presenting multi-component semiconductors or coupled semiconductors (composite, tandem, heterostructures, etc.). Additionally, in order to enhance certain properties these materials have been coupled with metals nanoparticles or doped with other metal ions. The MOS exhibit a multitude of morphologies such as: rods, stars, flowers, cone, porous or dense films, etc. This mini review will consider only four metal oxides (TiO_2_, SnO_2_, ZnO, and WO_3_) as representative for biosensor applications. Many other papers which are not included here have the potential to contain highly innovative work. A summarized data collection containing the four metal oxides is presented in Table 1.

**Table 1:** Representative studies on metal oxide semiconductors used in biosensors.

**Synthesis method/morphology**	**Target detecting substance**	**Testing method/LOD**	**References**
**TiO**_**2**_			
Dip-coating/Porous thin film	microRNA	Photocurrent/0.13 fM	Wang M. et al., 2019
	Heme	Photocurrent/19 μM	Çakiroglu and Özacar, 2019
	Glucose	Amperometric/0.7 μM	Rajendran et al., 2018
Dip-coating/nanorods	Glucose	CV/0.002 mM	Yang et al., 2014
Solvothermal/nanosheets		Photocurrent/0.01 mM	Liu P. et al., 2017
Hydrothermal/nanotube		DPV/0.5 μM	Zhu et al., 2015
Doctor blade/porous film	H_2_O_2_	CV/1.65 μM	Wu et al., 2018
Hydrothermal/microsphere		Amperometric/10 nM	Liu H. et al., 2017
Anodization/nanotubes	H_2_O_2_	Amperometric/0.08 μM	Kafi et al., 2011
	Cholesterol	CV/0.05 μM	Khaliq et al., 2020
	Cancer cells	Impedance/40 cells/mL	Safavipour et al., 2020
**SnO**_**2**_			
Precipitation/nanoparticles	L-cysteine	Chronoamperometric/ 0.03 μM	Dong and Zheng, 2014
Sonication/nanoparticles	Methyl parathion Carbofuran	CV/ 5 x 10^−14^ M 5 x 10^−13^ M	Zhou et al., 2013
Thermal evaporation/nanowires	H_2_O_2_	Impedance/0.8 μM	Li et al., 2010
Microwave irradiation/nanoparticles		DPV/43 nM	Lavanya et al., 2012
Electrospinning/nanowires	Glucose	Amperometry/1.8 μM	Alim et al., 2019
	Acetaminophen pHydroxyacetophenone	DPV/ 0.086 μM 0.033 μM	Hu et al., 2019
Physical vapor deposition/nanobelt	Cardiac troponin	Fluorescence microscopy/100 pM	Cheng et al., 2011
Hydrothermal/nanosheets	Amyloid β-protein	Photocurrent/ 0.17 pg/mL	Wang et al., 2018
**ZnO**			
Chemical bath deposition/nanostars	microRNA-21	CV/18.6 aM	Zhang et al., 2019
Chemical bath deposition/nanoparticles	Zika virus	CV/1.00 pg/mL	Faria and Mazon, 2019
Hydrothermal/nanocones	Dopamine	CV/0.04 μM	Yuea et al., 2020
Hydrothermal/nanorods	Phosphate	CV/0.5 μM	Ahmad et al., 2017
	G Imunoglobuline	DPV/0.03 ng/mL	Dong et al., 2017
	Glucose	DPV/1.0 μM	Zong and Zhu, 2018
Hydrothermal/nanoparticles	Glucose	CV/50 μM	Lei et al., 2011
**WO**_**3**_			
Simple casting/nanowires	Nitrite	Amperometry/ 0.28 μM	Liu et al., 2015
Hydrothermal/nanoparticles		CV/5 μM	Santos et al., 2016
Simple reversible redox/nanosheets	Epididymal protein 4	Colorimetric/ 1.56 pg/mL	Zhang et al., 2020
Ultrasonic/nanosheets	Xanthine	Colorimetric/ 1.24 μmol/L	Li et al., 2019
Hydrothermal/Flower-like	Aflatoxin B1	Photoelectrochemical/0.28 pg/mL	Feng et al., 2018
Hydrothermal/nanorods	Bisphenol A	DPV/0.028 μM	Zhou et al., 2017
Hydrothermal/nanocomposite	Cardiac biomarker Troponin I (cTnI)	DPV/0.01 ng/mL	Sandil et al., 2018

### TiO_2_-based Biosensors

TiO_2_ is an n-type semiconductor considered as a key material in many applications like photocatalysis, biosensors, photovoltaics, or energy storage due to his properties such as high chemical stability, biocompatibility, morphological versatility, etc.

Dip-coating technique was employed to obtain TiO_2_ films serving as sensors for microRNA (Wang M. et al., 2019), heme (Çakiroglu and Özacar, 2019), or glucose (Rajendran et al., 2018). The microRNA sensor is based on black TiO_2_ deposed on indium tin oxide (ITO) substrate and improved with Au nanopoarticles. The semiconductor was functionalized with histostar antibodies and based on photocurrent measurements the LOD was established at 0.13 fM. Photocurrent was used for LOD evaluation of heme using TiO_2_/ITO sensitized with CdS quantum dots, and the result was 19 μM. The glucose detector based on TiO_2_ film was functionalized with glucose oxidase (GOx) and the LOD was 0.7 μM. A better LOD value (0.5 μM) in glucose detection was obtained by replacing the TiO_2_ films with TiO_2_ nanotubes (Zhu et al., 2015). The nanotubes were developed using the hydrothermal method and functionalized with GOx. TiO_2_ nanorods (Yang et al., 2014) and nanosheets (Liu P. et al., 2017) were tested as glucose sensors after functionalizing with GO. Better LOD was obtained for nanorods morphology (0.002 mM) comparing with nanosheets (0.01 mM) mostly due to higher surface coverage, which was 3.32 × 10^−11^ mol/cm. Concluding, the LOD depends mostly on the active surface, which explains why nanotubes give better results compared with TiO_2_ films.

TiO_2_ was used for H_2_O_2_ detection in the form of microspheres (Liu H. et al., 2017) or macro-mesoporous film (Wu et al., 2018). The TiO_2_ microspheres with a bag-like structure were hydrothermally obtained and functionalized with hemoglobin (Hem). The amperometric measurements indicate a LOD of 10 nM. This value is considerably lower compared with TiO_2_ mesoporous films (1.65 μM LOD) obtained by doctor blade technique and functionalized with horseradish peroxidase (HPOx).

Anodization of titanium was intensively used to obtain TiO_2_ nanotubes for H_2_O_2_ (Kafi et al., 2011), cholesterol (Khaliq et al., 2020) and breast cancer cell (Safavipour et al., 2020) detection. When the TiO_2_ nanotubes were functionalized with Hem the LOD evaluated by amperometry was 0.08 μM. Better LOD value was obtained for cholesterol detection (0.05 μM) based on a non-enzymatic approach to the oxidation process. Finally, TiO_2_ nanotubes were functionalized with human mucin-1 aptamers, inducing sensitive electrochemical detection of breast cancer cells (MCF-7). In this case the 40 cells/mL LOD represent an encouraging result for future biomedical TiO_2_ application in breast cancer detection.

### SnO_2_-based Biosensors

Due to features such as high surface area, good biocompatibility, nontoxicity, excellent chemical stability, and catalytic activity, SnO_2_ was used in many applications such as light energy conversion, biosensors, smart windows, and electrochemistry. Tin oxide is an n-type semiconductor with a wide band gap of 3.8 eV and rutile structure (see Figure 1).

SnO_2_ nanoparticles were synthesized by precipitation (Dong and Zheng, 2014), sonication (Zhou et al., 2013) and microwave irradiation (Lavanya et al., 2012). By using precipitation method, the SnO_2_ nanoparticles have an average diameter of 4 nm and were used for L-cysteine detection. In order to employ the chronoamperometric detection method, the SnO_2_ surface was coated with multiwall carbon nanotubes giving a LOD of 0.03 μM. The SnO_2_ nanoparticles obtained by sonication methods were used for pesticide detection based on acetylcholinesterase as a functionalize agent. The LOD evaluated by cyclic voltametry (CV) was 5 × 10^−14^ for methyl parathion and 5 × 10^−13^ for carbofuran. The microwave irradiation method was employed to obtain SnO_2_ nanoparticles with application for H_2_O_2_ detection. SnO_2_ surface was functionalized with HPOx and, based on differential pulse voltammetry (DPV), a LOD of 43 nM was obtained.

An H_2_O_2_ sensor was developed using SnO_2_ nanowires synthesized by the thermal evaporation method (Li et al., 2010). Using the same functionalizing molecule as SnO_2_ nanoparticles, the LOD measured by CV was 0.8 μM. SnO_2_ nanowires were also obtained by the electrospinning method (Alim et al., 2019) for glucose amperometric detection. In this case the functionalizing procedure was done with both HPOx and GOx, giving a LOD of 1.8 μM. SnO_2_ nanobelts (Cheng et al., 2011) and nanosheets (Wang et al., 2018) were obtained by physical evaporation deposition, respectively using hydrothermal methods. The nanobelts functionalized with D-biotin molecules were successfully used as a troponin I detector, a protein marker for myocardial infarction (100 pM LOD). The nanosheets were used for amyloid β-protein (Aβ) detection after a previous functionalizing procedure with anti-Aβ antibody. Based on photocurrent measurements the LOD value was 0.17 pg/mL, considered as promising for applications in the detection of disease-related biomarkers.

### ZnO-based Biosensors

ZnO is a direct wide band gap semiconductor which under UV radiation exhibits n-type conductivity. During the crystallization forms a hexagonal wurtzite structure (see Figure 1) which has particular piezoelectric properties based on noncentrosymmetric crystal structures. The major part of the ZnO synthesis procedures are wet techniques. Compared with tin oxide, ZnO has a better binding ability with biological entities, which is a prerequisite for future biosensor applications in medicine. Due to its nontoxicity and compatibility with human skin, ZnO can be adapted as a permanent human sensor in chronic diseases such as diabetes.

Chemical bath deposition (CBD) has been used (Zhang et al., 2019) to obtain ZnO nanostars for detecting microRNA-21 in cancer cells. Previously, the surface was functionalized using thiol-modified hairpin and hybridization chain reactions, considering the development of electrochemiluminescence (ECL) biosensors. The LOD was evaluated at 18.6 aM, which makes this material a good candidate for clinical bioassay. The same technique was also employed (Faria and Mazon, 2019) to develop ZnO nanoparticles for detection of Zika virus in undiluted urine. The Zika virus is transmitted through mosquito bites and gives symptoms such as headaches, arthralgia, myalgia, or conjunctivitis (Faria and Mazon, 2019). The ZIKV-NS1 antibody was immobilized using cystamine and glutaraldehyde on the ZnO nanoparticles. The LOD was evaluated using CV and the result was 1.00 pg/mL. This MOS biosensor can be used in early detection of the Zika virus.

Another technique that has been extensively used for ZnO synthesis with biosensing application is the hydrothermal procedure. Both ZnO nanorods (Zong and Zhu, 2018) and nanoparticles (Lei et al., 2011) hydrothermally obtained were used in biosensors for glucose detection. The ZnO nanorods where functionalized with GOx by simple immersion and the LOD via DPV was 1.0 μM. These results are significantly better compared with ZnO nanopowder functionalized with GOx, where LOD was 50 μM. ZnO nanorods where hydrothermally obtained and used as sensors for phosphate (Ahmad et al., 2017) and G Imunoglobuline (Dong et al., 2017) detection. For phosphate detection the ZnO was functionalized with pyruvate oxidase by immersion, and the LOD was 0.5 μM. In order to develop a G Imunoglobuline sensor with 0.03 ng/mL LOD, the ZnO surface was functionalized with myoglobin by immersion and cold drying. ZnO nanocone arrays were developed using the hydrothermal technique (Yuea et al., 2020) for dopamine detection. The nanocones were functionalized using Au nanoparticles with carboxyl groups obtaining a sensor with high sensitivity (4.36 μA/μM) and low LOD (0.04 μM).

### WO_3_-based Biosensors

WO_3_ is an n-type semiconductor with a band gap of 2.8 eV and a versatile crystalline structure varying from cubic to octahedral, depending on the synthesis temperature. High surface to volume ratio WO_3_-based materials can be developed using physical and chemical techniques with well-controlled dimensionality, sizes, and crystal structure for sensors research.

A WO_3_ nitrite-based sensor was prepared by a simple casting (Liu et al., 2015) and hydrothermal (Santos et al., 2016) methods. In the first case WO_3_ has nanowire morphology and was functionalized with hemoglobin, while WO_3_ nanoparticles were obtained and functionalized with cytochrome c nitrite reductase using the hydrothermal procedure. The LOD value in the case of WO_3_ nanowire is significantly lower (0.28 μM) compared with WO3 nanopowder (5 μM) underlining the significance of semiconductor morphology, synthesis and functionalizing procedures. The hydrothermal method has been used to produce WO_3_ sensors with different morphologies: flower-like for aflatoxin B1 (Feng et al., 2018), nanorods for bisphenol A (Zhou et al., 2017), and nanosheets for cardiac biomarker Troponin I (Sandil et al., 2018). The WO_3_ flower-like morphology was functionalized with bovine serum albumin and the LOD corresponding to aflatoxin B1 was very low (0.28 pg/mL). The nanorods were doped with Na^+^ ions, giving a LOD value of 0.028 μM. Finally, the nanosheets were functionalized with 3-aminopropyl tri-ethoxy saline for the activation of amino groups and the LOD value was 0.01 ng/mL. In the case of WO3-based biosensors the nanosheets morphology gives a better LOD for cardiac biomarker Troponin I compared with the flower-like morphology used for alfatoxin B1 detection.

Other techniques were used to obtain WO_3_ nanosheets, such as simple reversible redox (Zhang et al., 2020) and ultrasonic exfoliation (Li et al., 2019) processes. Using the reversible redox process makes it possible to develop WO_3_ nanosheets with an average width of 150 nm, and LOD for human epididymal protein 4 detection of 1.56 pg/mL. Ultrasonic exfoliation of bulk WO_3_ in water allows the preparation of WO_3_ nanosheets in the range of 20 to 40 nm. These nanosheets were functionalized with 3, 3′, 5, 5′-tetramethylbenzidine and the LOD for xanthine in urine (based on colorimetric evaluation) was 1.24 μmol/L.

## Conclusions

Metal oxides are considered as versatile materials that can be successfully integrated in biosensor technology. Based on features such as chemical stability, light excitation/light conversion, and high surface-to-volume ratio, these materials are highly competitive in the biosensors market. This mini review has outlined that the biosensors' LOD depends on many parameters such as: morphology (active surface), functionalizing molecule, evaluation procedure, and detecting target. The same material with similar morphology and detecting targets can give different LOD depending on surface functionalization and evaluation procedures. Even if most of the biosensors are used for glucose (H_2_O_2_, uric acid) there are encouraging results for cancer cell or virus detection. Recent advancements indicate a promising future for MOS in applications such as skin bioelectronics, neural interfaces, and smart biosensing devices. However, much effort is required to overcome important issues related to optimizing organic/inorganic interface compatibility, the enzyme electrochemistry at the MOS interface, and LOD improvement. To achieve this goal, facile MOS synthesis technologies allowing good interface control must be implemented.

## Author Contributions

IŞ was responsible for article database regarding TiO_2_, SnO_2_, and WO_3_, including Table 1. AE has coordinated the writing, introduction, biosensors mechanism (including Figure 1), and article database for ZnO.

## Conflict of Interest

The authors declare that the research was conducted in the absence of any commercial or financial relationships that could be construed as a potential conflict of interest.
